# An associative account of collective learning

**DOI:** 10.1098/rsos.241907

**Published:** 2025-03-26

**Authors:** Matthew Gildea, Cristina Santos, Federico Sanabria, Takao Sasaki

**Affiliations:** ^1^ Department of Psychology, Arizona State University, Tempe, AZ 85287, USA; ^2^ Universidad Anahuac Cancun, Cancun, QR 77565, Mexico; ^3^ Odum School of Ecology, University of Georgia, Athens, GA 30602, USA; ^4^ Brain and Cognitive Sciences, University of Rochester, Rochester, NY 14627, USA

**Keywords:** collective learning, associative learning, mathematical modelling, groups, reversal learning, collective intelligence

## Abstract

Associative learning is an important adaptive mechanism that is well conserved among a broad range of species. Although it is typically studied in isolated animals, associative learning can occur in the presence of conspecifics in nature. Although many social aspects of individual learning have received much attention, the study of collective learning—the acquisition of knowledge in groups of animals through shared experience—has a much shorter history. Consequently, the conditions under which collective learning emerges and the mechanisms that underlie such emergence are still largely unexplored. Here, we develop a parsimonious model of collective learning based on the complementary integration of associative learning and collective intelligence. The model assumes (i) a simple associative learning rule, based on the Rescorla–Wagner model, in which the actions of conspecifics serve as cues and (ii) a horse-race action selection rule. Simulations of this model show no benefit of group training over individual training in a simple discrimination task (A+/B−). However, a group-training advantage emerges after the discrimination task is reversed (A−/B+). Model predictions suggest that, in a dynamic environment, tracking the actions of conspecifics that are solving the same problem can yield superior learning to individual animals and enhanced performance to the group.

## 1. Introduction

Learning is the process by which organisms acquire knowledge about their environment and their relation to that environment through experience [1,2]. One of the simplest forms of learning is associative learning, a dynamic process in which organisms learn about the correlation between stimuli and between their own actions and their outcomes [3–5]. Without associative learning, an organism would not be able to develop efficient strategies to find important resources, such as food and shelter, or avoid threats, such as predators, in their dynamic habitats. The importance of associative learning for evolutionary fitness is reflected in its conservation among species, ranging from *Caenorhabditis elegans* [6] and tardigrades [7] to macaques [8] and humans [9].

Various models of associative learning have been proposed since the middle of the twentieth century [10–12]. Over the last few decades, these models have become increasingly sophisticated, incorporating key psychological constructs such as attention [13] and causal attribution [14], to account for challenges to earlier, simpler models [15]. An interesting feature of learning organisms, overlooked in these previous associative learning models, is that organisms may be organized into larger ensembles that may themselves have learning capabilities. This collective learning, in which groups of animals acquire knowledge through shared experience, has been investigated primarily in the human sciences, including sociology, psychology, anthropology and education [16–18]. However, non-human social species also form groups and repeatedly perform the same tasks together [19], providing opportunities to improve their collective performance, such as when foraging [20–22], by learning together. Yet, the field of animal learning has mainly focused on individual learning and has directed little attention to associative learning by multiple entities. As a result, researchers typically study the associative learning abilities of social animals in isolated conditions [23–28]. Furthermore, collective learning research in humans has been accounted for mostly in terms of sophisticated cognitive mechanisms, such as mental state attribution (understanding other persons’ beliefs, feelings and intentions) [17,18,29]. It is therefore little known if and how non-human animal groups attain synergetic and advantageous performance by learning together.

Interest in social factors involved in individual animal learning is not new. For instance, various species have been shown to engage in vicarious learning, in which individuals learn associations not directly experienced by themselves, but instead experienced by conspecifics, through observation or direct communication [30–33]. Indirectly learnt information can then spread throughout populations and generations [34]. Additionally, animals engage with social cues, such as when visual and olfactory cues that signal the presence of a familiar same-sex conspecific; honeybees [31] and socially isolated rats [35] learn to approach those cues. These forms of social learning, which typically focus on how focal individual animals learn from other group members [36,37] have revealed important learning processes within groups [21,38,39]. However, collective learning is distinct from them: collective learning is the acquisition of social and non-social information *as a group* that may emerge from dynamic learning processes at the individual level over time [19].

We propose a framework to study collective learning that integrates two well-established fields of inquiry, collective intelligence and associative learning. Collective intelligence research focuses on the capacity of groups of animals to solve problems with more efficiency and efficacy than any of the individuals that constitute those groups [40]. The field of collective intelligence, however, typically neglects how dynamic learning processes that unfold at the individual level may give rise to enhanced group performance [19,41]. Conversely, associative learning research focuses on individual processes but often overlooks how such processes unfold in the context of other learning conspecifics, a more natural setting for many social organisms, including humans. The integration of these fields may unveil how groups change their behaviour based on experience and how such changes emerge from relatively simple processes occurring in individuals.

In this article, we first briefly review collective learning research. Building on findings from this research, we introduce a novel model of collective learning from simple associative mechanisms that operate at the level of individual organisms. We then investigate the conditions under which, according to the model, collective learning emerges. Finally, we discuss the advantages of our proposed model over similar social learning models.

## 2. Collective intelligence and learning

In many taxa, from bacteria to humans, individuals cooperate to evaluate their environment and make collective decisions [42–46]. By combining multiple assessments, these groups attain more precise estimates and make more accurate decisions than solitary animals, a phenomenon known as collective intelligence [47–49]. For example, when group members independently estimate a quantity (e.g. humans guessing the number of marbles in a jar), the group’s average estimate is often closer to the actual value than most of the individual estimates [50–52]. Similarly, schools of fish [45,53] and colonies of ants [54] can better distinguish between stimuli than individuals, and flocks of homing pigeons take more efficient routes than solitary birds [55]. Condorcet’s jury theorem and the central limit theorem mathematically show how groups reach better decisions than solitary individuals [56]. Accounts of collective intelligence based on these theorems assume that collective decisions are simple aggregations of individual decisions—average values or majority opinions. However, animal collective decision-making in nature often relies on decentralized mechanisms in which interactions and feedback bring the group to consensus [57–59].

Collective learning may be conceived as a form of collective intelligence because individuals in the group collectively acquire information and retrieve it in the future and, by doing so, outperform lone individuals. Collective learning is therefore sometimes confused with other forms of experience-driven collective intelligence. To illustrate this distinction, consider Flack & Biro’s [60] demonstration that pairs of pigeons acquire homing routes faster and with lower variability than individual pigeons. It is conceivable that pairs of pigeons perform better because the presence of a conspecific facilitates, in some way, the acquisition of navigational information through experience. This would constitute collective learning. It is also conceivable, however, that pairs perform better because pigeons have some tendency to approach each other, bringing their routes closer to a more efficient average by combining routes based on the central limit theorem [55]. This is not collective learning because, despite experience (the sight of a conspecific) yielding a change in performance, it does not change the parameters of the learning process.

The distinction between collective learning and collective intelligence suggests that demonstrations of the former require more than just experience-driven improvements in group performance. Even though various theoretical models have been proposed to explain how collective intelligence may emerge from collective learning, to the best of our knowledge, carefully designed experiments to show that group performance improvements reflect substantial changes in learning parameters have not yet been conducted.

## 3. Previous collective learning models

Falcón-Cortés *et al*. [61,62] offer one of the simplest collective learning models in the literature. In this model, agents acquire optimal performance in a simulated foraging task by each occasionally repeating past actions of their own (memory) and of other agents (communication). This is a minimal learning algorithm, where learning consists simply of a memory update and probabilistic information transfer between foragers. No associative learning takes place in this model, as actions are encoded and retrieved from memory according to fixed rules.

Kao *et al*. [63] present a more complex collective learning model that is based on associative learning. In their model, agents vote for one of various actions (corresponding to distinct cues), each of which yields a reward with some reliability. The action with the most votes is selected as a group decision, and each agent obtains the reward. The strength of the association between the cue that corresponds to the selected action and the reward is then independently updated for each agent according to a rule similar to the Rescorla–Wagner [64] rule


(3.1) ΔV=β(λ−ΣV),

where Δ*V* is the change in associative strength of the selected cue between trials *t* and *t* + 1, *β* is the rate of learning, *λ* indicates whether a reward was obtained (non-zero) or not (zero) and Σ*V* is the sum of the associative strengths of all cues present on a given trial. The probability that an agent votes for an action is a function of the strength of the association between its corresponding cue and the reward. When trained according to this model, groups are superior to individuals in obtaining rewards.

Like Falcón-Cortés *et al*.’s [61,62] model, Kao *et al*.’s [63] model has three components: memory, action selection and communication. However, memory in Kao *et al.*’s model is not of the actions selected before but of their association (via their corresponding cue) with a reward. Actions are therefore governed not only by the momentum of past actions (one’s own or someone else’s) but also by the meaning that they directly acquire through experience. Furthermore, communication among agents in the model does not involve direct access to the memory of the other agents (as in Falcón-Cortés *et al*.’s model) but is instead implemented indirectly through the forced selection of actions via consensus decision.

Memory, action selection and communication thus appear to be critical components of any collective learning model. The model we propose—the conspecific cue model or CCM—incorporates these three components in a manner similar to the previous models described above, but with a key difference in the communication component. In our model, communication is implemented through the signalling function of the actions of other agents. That is, actions from other agents may be associated with reward and thus function as cues. In our model, unlike the model developed by Kao *et al*., consensus decision is not built as a communication feature, but it may instead emerge from the collective learning process itself. We describe our model in detail below.

## 4. Conspecific cue model: collective learning emerging from individual learning

The essence of CCM is that collective learning emerges from individual agents learning simple associations between the actions of conspecifics and relevant outcomes. A key feature of CCM is that those conspecifics also learn from the actions of other individuals, giving rise to a collective change in behaviour. The model, therefore, has two components: (i) a learning component, in which cue-driven reward expectations are updated based on feedback and (ii) an action-selection component, in which such expectations are translated into actions that, in turn, serve as cues for other individuals. The communication component of CCM is folded within the learning component because the behaviour of conspecifics constitutes reward-informative cues.

### 4.1. Learning

One of the earliest, simplest and more versatile associative learning models is the Rescorla–Wagner model [64]. The Rescorla–Wagner model explains a broad range of learning phenomena using only a few assumptions on how stimuli (cues) are processed as predictors of other stimuli (rewards). Just like Kao *et al*.’s (2014) model, CCM assumes that individual animals learn the association between stimuli following a simplified version of the Rescorla–Wagner rule (equation (3.1)). The adoption of this learning rule is, however, a weak assumption in CCM, as the Rescorla–Wagner rule may be substituted with any other feedback rule that updates the strength of associations between actions and rewards.

**Figure 1.**
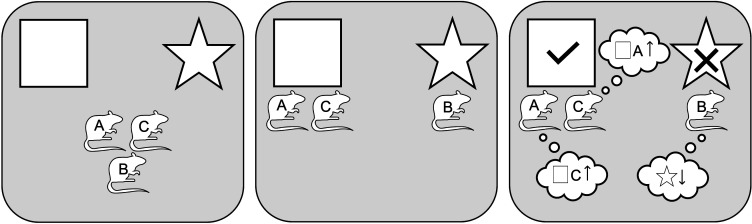
Schematic representation of the CCM of collective learning. *Left panel*: three rats are presented with two options, square versus star. *Centre panel*: following an action-selection rule (equation (4.1)), two subjects, rats A and C, choose the square and one subject, rat B, the star. *Right panel*: only the square was baited with reward. A learning rule (equation (3.1)) strengthens the association between the square and the reward for rat A (and C), and between the choices of rat C (and A) and the reward; for rat B, the rule weakens the association between the star and the reward and does not change the association between rat A or C and reward.

One unique feature of CCM is that the actions of conspecifics are cues that can be associated with reward. Consider the following situation (figure 1): a group of rats must choose between two options, each signalled with a different visual stimulus (square versus star), but only one (square) is always baited with a reward. At the onset of training, the rats have not learnt yet which option is baited, so they choose randomly. For those that chose the baited option, the association between the reward and the corresponding cue (square) is strengthened according to equation (3.1), so that they are more likely to select that cue in a future trial. However, if the association between the actions of other ‘successful’ rats and the reward is also strengthened, then these actions can also serve as predictive cues, guiding behaviour and increasing the probability of choosing the same option as the successful rats. To the extent that successful rats are more likely to visit the baited option in the future, tracking them (as any other cue) would yield more visits to the baited option in future trials.

### 4.2. Action selection and execution

For associative strength to guide behaviour, it must be translated into actions. Whereas the Rescorla–Wagner model and many other learning models do not precisely specify the relation between learning and action, CCM must do so, because the actions of other individuals are key inputs to the learning process. In the example from figure 1, the rat that acts first must do so solely based on non-social stimuli already present (square and star); other rats may then use the actions of preceding rat or rats to choose between alternative actions. Various rules may be conceived to determine which group member acts first [65]. Here we propose that response times emerge from a simple ‘horse-race’ action selection rule: the strength of the association between the reward and each possible action (via cue–reward association) determines the speed at which the action is selected; the first action selected suppresses all alternative actions, with the individual making the quickest selection acting first.

In the example of figure 1, there are two possible actions, ‘go square’ or ‘go star’, each linked to a cue, square and star, respectively. Following a small modification of Ghirlanda’s suggestion [66], the probability *p*(*j*) that an individual selects action *j* at each time step since trial onset is a mixture of the fixed probability *m* of selecting it by non-associative exploration and the cumulative exponential function of the sum of the associative strength of all cues—non-social and social—that correspond to that action (Σ*V*(*j*); see equation (3.1))


(4.1) $$p\left(j\right)=m+\left(1-m\right)\left(1-e^{-c\sum V\left(j\right)}\right),$$

where *c* is a positive scale constant. Each agent samples among actions in consecutive time steps until at least one action is selected. If only one action is selected at a time step, that action is executed. If more than one action is selected, all non-selected actions are rejected, and sampling continues only with the selected actions until only one action is selected.

### 4.3. The conspecific cue model algorithm

**Figure 2.**
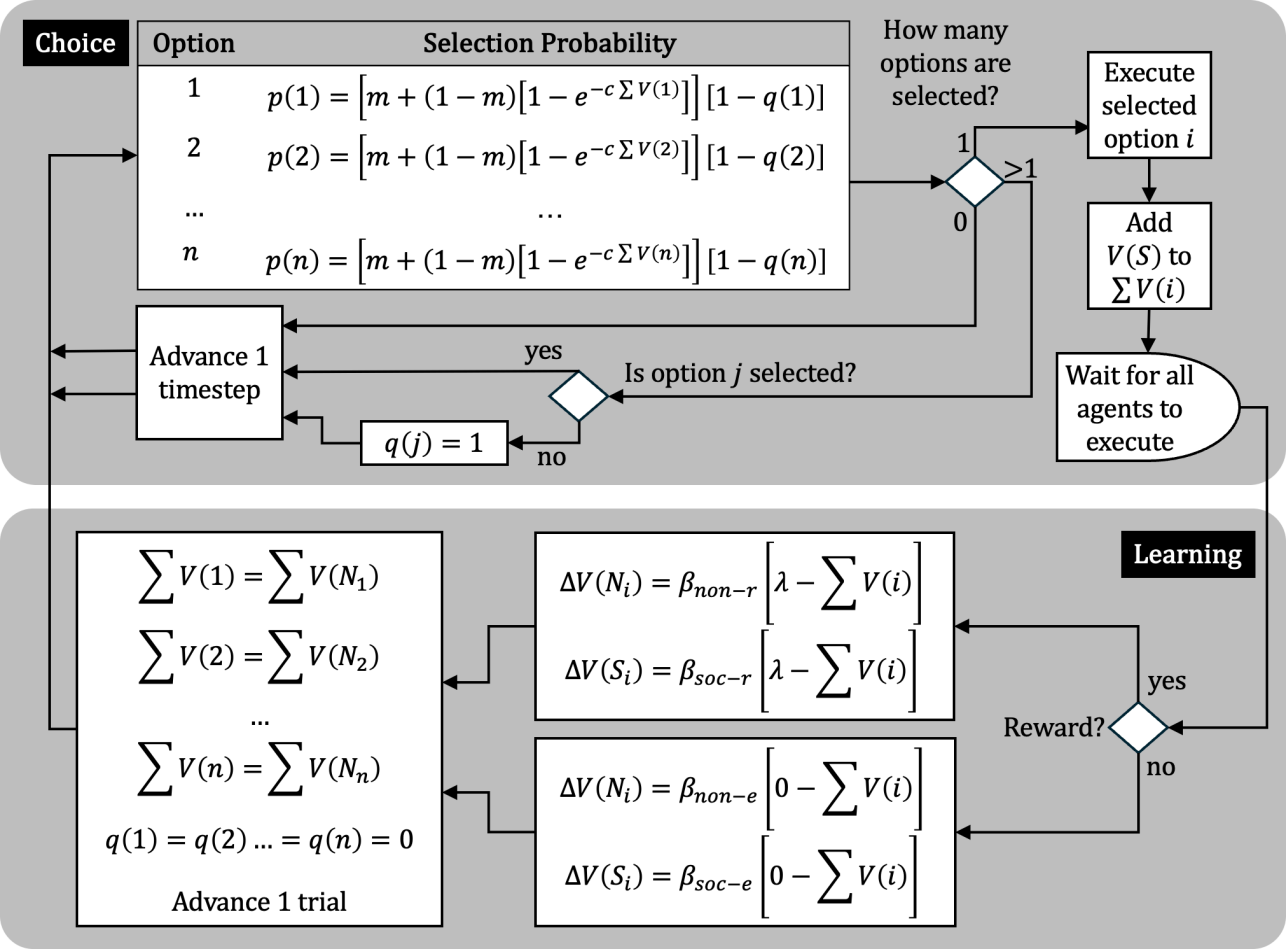
Algorithmic representation of CCM. *Choice component*: actions are selected from *n* options with probability *p*(*j*). Selection of option *j* depends on the probability of non-associative exploration (*m*), the sum of the corresponding non-social and social cues [Σ*V*(*j*)], a scale constant (*c*) and a binary control variable that indicates whether *j* has already been rejected [*q*(*j*)]. The selection process is repeated until a single option is selected and then executed. *Learning component*: once all agents execute an option, for each agent that executed *i*, the associative strength of non-social and social cues [*V*(*N_i_*) and *V*(*S_i_*)] of the selected option *i* increase toward *λ* if it was reinforced; otherwise, they decrease toward zero. Agents are then removed from every option, so every Σ*V*(*j*) becomes simply Σ*V*(*N_j_*), and binary control variable *q* is reset, before advancing to the next trial.

Figure 2 shows a general algorithmic representation of CCM for any individual agent. It starts in the top-left corner, in the choice component. At the onset of each choice trial, the agent is presented with *n* choice options. The probability of selecting any option *j* is *p*(*j*). This probability is a function of the probability of non-associative exploration (*m*), the sum of the associative strengths of all cues associated with *j* [Σ*V*(*j*)] and a scaling factor *c*. At this first time step of each trial, all *q*(*j*), a parameter determining whether an option has been rejected, are set to zero. With larger *m*, all options become similarly—and highly—likely to be selected; with smaller *m*, selection depends more on the associative strength of all the cues, non-social (e.g. squares and stars) and social (actions of other agents), linked to each option.

With these probabilities, the agent may select between zero and *n* options. If no option is selected, the algorithm advances one time step, and a selection is made again. If more than one option is selected, then *q* = 1 for all options that were not selected; the algorithm advances one time step, and a selection is made again. The binary control variable *q*(*j*) effectively excludes non-selected options from being considered in subsequent time steps. This loop eventually settles in the selection of just one option, which is the executed option—e.g. the rat approaches the square.

Every agent that has not yet executed an option can see other agents execute theirs. Therefore, once an agent executes option *i*, this action becomes a cue for that option, adding its associative strength, *V*(*S*), to Σ*V*(*i*). On the first trial, it may be assumed that *V*(*S*) = 0 (i.e. the actions of other agents are not associated with reward) for all the options, but *V*(*S*) is expected to change over trials (more on this below). A key feature of *V*(*S*) is that it may vary between agents and social cues. More formally, *V*(*S*) is a square hollow matrix with agents as rows and social cues as columns. For a scenario with three agents, A, B and C, *V*(*S*) may thus be represented as


(4.2) $$V\left(S\right)=\left(\begin{array}{ccc} 0 & v_{A,B} & v_{A,C}\\ v_{B,A} & 0 & v_{B,C}\\ v_{C,A} & v_{C,B} & 0 \end{array}\right),$$

where *v_row_*_,*col*_ is the strength of the association, for agent *row*, between the actions of agent *col* and the reward.

Thus, for any agent A that is selecting between options, once agent B executes option *i*, Σ*V*(*i*) increases by *v*_A,B_. In the example of figure 1, if rat A went to the square before rats B and C, and the actions of rat A had a positive association with reward for rats B and C (i.e. *v*_B,A_ > 0 and *v*_C,A_ > 0), the execution of this option would increase Σ*V*(‘go square’) for rats B and C, increasing their probability of selecting it.

The learning component starts once all agents execute an option. For those that receive a reward, the associative strength of every non-social and social cue present at the executed option *i* [*V*(*N_i_*) and *V*(*S_i_*), respectively] converges with *λ* (an arbitrary non-zero value) according to equation (3.1). The algorithm allows different rates of reward learning for non-social and social cues (*β_non-r_* and *β_soc-r_*, respectively). For those that do not receive a reward (extinction), analogous changes in *V*(*N_i_*) and *V*(*S_i_*) are implemented, converging instead with zero at two other rates (*β_non-e_* and *β_soc-e_*).

Once the associative strengths of non-social and social cues linked to option *i* are updated, trial parameters are reset. First, agents are removed from their executed options before the onset of the next trial, so the sum of the associative strengths of all cues in each option becomes the sum of the associative strength of just the non-social cues in that option. Second, all *q*(*j*) are reset to zero. Finally, a new choice trial starts.

### 4.4. Model simulations

**Figure 3.**
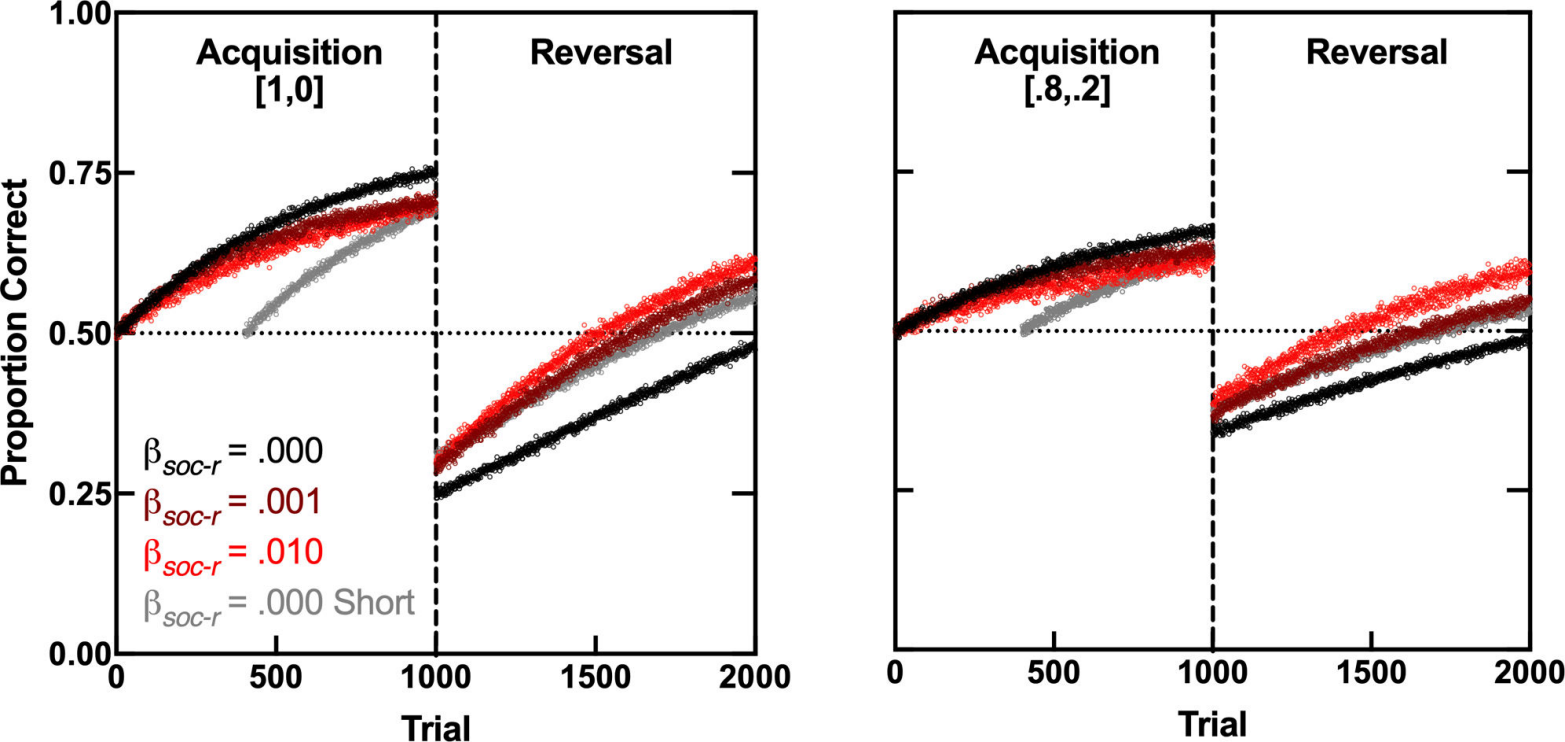
Simulations of CCM under various scenarios. Curves trace 1000 runs of 10 simulated agents, based on the algorithm in figure 2. The dashed vertical line separates two training phases: cue discrimination (acquisition) and its reversal. The dotted horizontal line indicates chance performance. Each curve was generated using different *β_soc-r_* and *β_soc-e_* values (*β_soc-e_* = *β_soc-r_* /2). Other model parameters were fixed: starting associative strengths (*V*) of social and non-social cues = 0; *β_non-r_* (learning rate for reinforced selection of non-social cues) = 0.001; *β_non-e_* (learning rate for extinguished selection of non-social cues) = *β*_*n*_*_on-r_* /2 = 0.0005; *λ* was unity; *m* = 0.05; *c* = 0.2. Note that, for the *β*_*s*_*_oc-r_* = 0.001 condition (dark red curve), the learning rate for social and non-social cues is the same. The two *β_soc-r_* = 0.000 only differ in the number of acquisition trials (600 versus 1000). The numbers in brackets indicate cue reliability, [*p*(*r*|corr), *p*(*r*|incr)]. See text for more details. See electronic supplementary material for simulation code.

Figure 3 shows three simulations of CCM over 600 or 1000 training trials of a simple two-stimulus discrimination (e.g. ‘go square’ is correct, ‘go star’ is not), followed by 1000 training trials of the reversed discrimination (e.g. ‘go star’ is correct, ‘go square’ is not). In the left panel, the correct action was always reinforced [*p*(*r*|corr) = 1] and the incorrect selection was never reinforced [*p*(*r*|incr) = 0]. In the right panel, cues were less reliable: *p*(*r*|corr) = 0.8 and *p*(*r*|incr) = 0.2. Each of 1000 simulation runs traces the mean proportion of correct actions of 10 simulated agents. Across simulations, the reward learning rate of social cues (*β_soc-r_*; see figure 2) was varied; the extinction learning rate of social cues (*β_soc-e_*) was set at half of *β_soc-r_*; other parameters were fixed [67]. Thus, setting *β_soc-r_* to zero (black and grey curves) was equivalent to training 10 agents individually.

The three simulations consisting of 1000 acquisition trials in figure 3 (*β_soc-r_* = 0.000, *β_soc-r_* = 0.001 and *β_soc-r_* = 0.010) show that, regardless of cue reliability, CCM predicts that individually trained agents learn a novel discrimination faster than group-trained agents; lower reliability simply flattens the learning curve. Thus, reducing the reliability of non-social cues is functionally similar to reducing their learning rates (*β_non-e_* and *β_non-r_*, not simulated). However, the model also predicts that group-trained agents learn the reversal of the discrimination faster than individually trained agents, particularly when the learning rate of social cues is high. Therefore, although CCM predicts an advantage of individual over collective learning in relation to novel stimuli, it predicts an advantage of collective learning—i.e. collective intelligence—when the meaning of those stimuli changes.

There are a few potential caveats to this interpretation of CCM predictions. The first one is that collective intelligence during reversal may be an artefact of the superior performance of individual learners at the end of the acquisition phase, which yields a lower performance at the onset of the reversal phase. The *β_soc-r_* = 0.000 shorter condition in figure 3 addresses this possibility. In this condition, the acquisition phase was conducted with individually trained agents for just 600 trials, so that their performance at the onset of the reversal phase was comparable to that of group-trained agents (*β_soc-r_* = 0.001 and 0.01). Reversal performance shows a collective-training advantage even under these conditions, particularly when cues are reliable (left panel).

**Figure 4.**
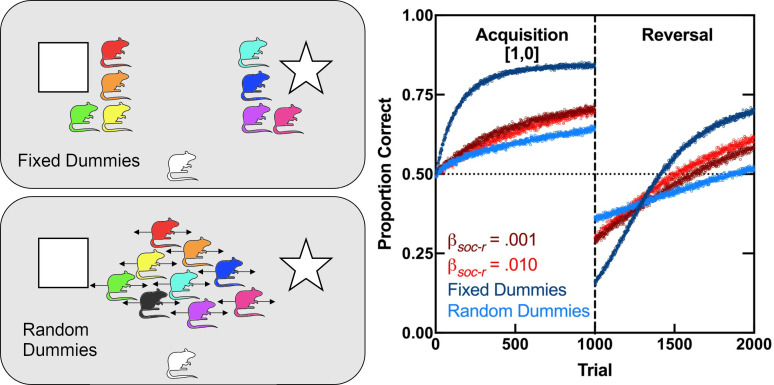
Simulation of CCM with fixed and random dummies. Left panels illustrate two alternative training conditions for an individual agent. In the *Fixed Dummies* condition (top), there are five redundant non-social cues in each option (shapes and dummy rats). In the *Random Dummies* condition (bottom), two non-social cues are always placed in the same option (shapes), whereas the other nine non-social cues are placed randomly with equal probability across options on each trial (dummy rats). The right panel shows simulated performance under these conditions. Simulations were conducted as in figure 3, left panel, except where indicated otherwise. The *β_soc-r_* = 0.001 and 0.010 conditions are included again for reference. In the *Fixed Dummies* condition, *β_non-r_* = 0.005, *β_non-e_* = 0.0025, *λ* = 0.2 and *c* = 0.2 (see equation (4.2) and text for rationale). In the *Random Dummies* condition, default non-social parameters were reinstated. In both conditions, performance of a single agent was tracked over 10 000 runs of the simulation.

Another potential caveat is that individually trained agents experience fewer cues on each trial than group-trained agents: whereas the former experience only non-social cues, the latter experience non-social and social cues. Fewer cues lower Σ*V*(*i*) and thus effectively lower the learning rate. To appreciate this effect, consider equation (3.1) when *K* cues, each with associative strength *V*(*j*), are present. This would replace Σ*V* with *KV*(*j*) in equation (3.1), which may also be expressed as


(4.3) ΔV(j)=βK[λ/K−V(j)].

Therefore, as *K* increases, each cue is expected to reach a lower reward learning asymptote (λ/*K*) faster (*βK*; see learning component in figure 2). The lower learning asymptote of each of multiple cues predicts the well-established overshadowing effect. It does not predict, however, a reduction in the probability of selecting their corresponding option, because that probability is a function of Σ*V*(*j*) = *NV*(*j*) (see choice component in figure 2). The faster reward learning of each of multiple cues, on the other hand, predicts a rapid rise of the probability of selecting and executing their corresponding option. Thus, the collective training advantage in reversal performance may simply reflect the larger number of cues involved in this condition.

The *Fixed Dummies* condition in figure 4 simulates this situation, training agents individually but with *n* = 5 redundant cues (shapes and dummies in top-left panel) on each option instead of just one. Its simulation was conducted according to figure 2, with parameters adjusted following equation (4.3) to make them comparable with the individual-training condition in figure 3 (e.g. *β_non-r_* = 0.001 with *n* = 1, so *β_non-r_* = 0.005 with *n* = 5). The simulation shows that, according to CCM, more cues—social or not—indeed facilitate acquisition and reversal learning, even when performance at the onset of this phase is inferior.

The learning advantage of having more cues emerges only when those additional cues—the dummies—always execute the same option. If, instead, the dummies execute a random option on each trial, the advantage turns into a disadvantage. This is shown in the *Random Dummies* condition (figure 4, bottom-left panel), in which agents are trained individually in the presence of two non-social cues (e.g. square and star), one assigned to the correct option and another to the incorrect option, and nine dummies that are assigned randomly to each option on each trial. The right panel of figure 4 shows that the uninformative dummies, according to CCM, undermine performance during both phases, acquisition and reversal.

Taken together, the simulations of figures 3 and 4 indicate that CCM can account for a collective learning advantage over individual learning in a relatively noisy environment, where dynamic non-informative cues obscure informative cues. Even if non-informative cues are removed, CCM shows that learning about ambiguous cues, such as those whose meanings have reversed, may benefit from collective training.

**Figure 5.**
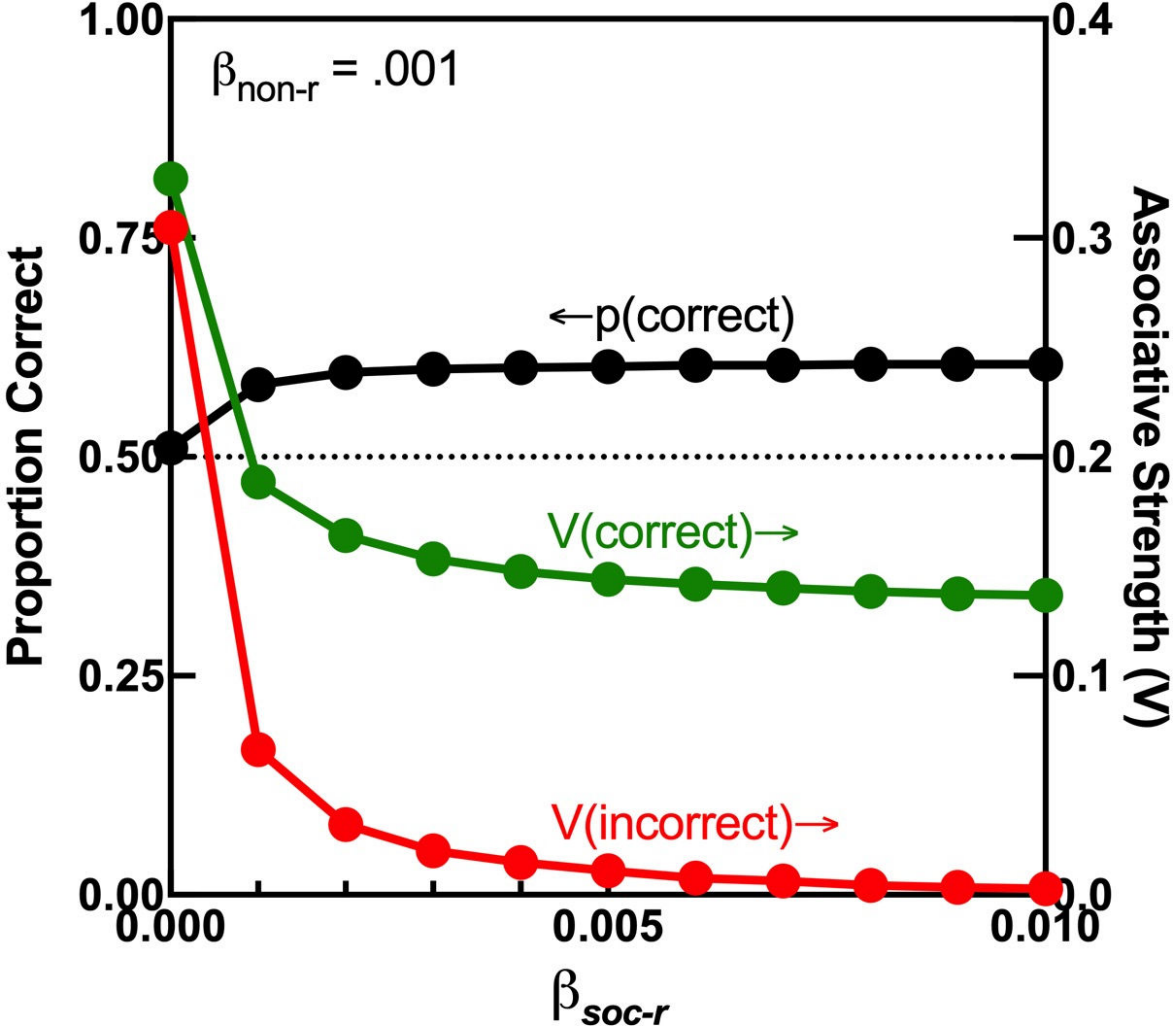
Individual performance and learning following reversal training. Curves trace the mean individual proportion of correct choices [*p*(correct), left *y*-axis] and associative strengths of non-social cues [*V*(correct) and *V*(incorrect), right *y*-axis], obtained with various social learning rates after the reversal training of 10 agents (*β_soc-r_*). To avoid differences in performance at the onset of the reversal phase, the acquisition phase was terminated when mean *p*(correct) > 0.75 over a moving window of 50 trials. Other simulation parameters were the same as in figure 3, left panel.

Nonetheless, in the strictest sense, figures 3 and 4 show that collective training enhances group performance but not necessarily individual learning. Individually trained agents may have learnt the correct option better than collectively trained agents, but the latter may outperform the former by following a small number of agents in the group that have learnt the task well. Figure 5 shows, however, that this is not the case. After a reversal training of 10 simulated agents, a social learning rate *β_soc-r_* of zero (individual training) and a small non-social learning rate (*β_non-r_* = 0.001) yield very similar associative strengths (*V*) for the correct and incorrect non-social cues, resulting in poor individual performance [*p*(correct)]. As *β_soc-r_* increases, the social cues overshadow the non-social cues, so *V*(correct) and *V*(incorrect) decline. However, *V*(correct) declines less than *V*(incorrect) with larger *β_soc-r_*, resulting in enhanced individual performance. Therefore, CCM predicts that collective training can yield not only better group performance but also better individual learning.

## 5. Non-collective learning models related to the conspecific cue model

The central assumption of CCM is that the behaviour of conspecifics may operate simultaneously as an informative cue for all animals in a learning situation. Other models of social learning appear to incorporate this assumption, but their aims are more ambitious, so they require further assumptions that may or may not be critical for collective learning to emerge. A good example of such a model is Lind *et al*.’s [68] social learning model. There is substantial overlap between their model and CCM: both assume a functional similarity of social and non-social cues, a simple learning rule for combining elements of compound cues, and a simple action rule for translating learning into behaviour. However, Lind *et al*.’s model is less concerned with collective learning (simultaneous group learning) than it is with other forms of social learning—in particular, with learning from already experienced agents. To account for this form of social learning, Lind and colleagues’ model assumes an innate higher salience of actions of experienced conspecifics via genetic predisposition. CCM may incorporate such salience differential in the form of a higher *β*_soc-r_, but, as shown in the simulations, it is not required.

Furthermore, unlike CCM, Lind *et al*.’s model is also concerned with social facilitation in the formation of complex behavioural sequences. In other words, whereas the CCM relies on a single assumption on learning (equation (3.1)), Lind *et al*.’s model must distinguish between Pavlovian and instrumental learning, innate and learned stimulus values—all combined into their A-learning theory [69]—and between actions performed by an agent and those perceived by a naive observer, among other assumptions that may limit its applicability to naturally social animals.

To address the problems within the scope of their model, Lind *et al*.’s [68] social learning model must grapple with what they call the correspondence problem: how does the observer establish a link between the behaviour of a conspecific (e.g. animal A eats an apple) and its own behaviour (observer B eats an apple)? For their simulations, they assume a non-zero value of unconditioned imitation but ultimately acknowledge that there may not be a general solution to the correspondence problem. Because CCM has a narrower aim, it does not need to assume the distinctions that Lind and colleagues do, and it does not need to address the correspondence problem. Animal A eating an apple is, for CCM, a cue that may acquire conditioned properties just as a light or a sound, by decreasing the prediction error for an outcome, with no further assumptions required.

## 6. Conclusion

Current theories of collective learning provide formal descriptions of how groups of animals learn and behave. However, we note at least two gaps in these theories. First, although some theories emphasize the functional similarity of social and non-social cues (e.g. [68]), they focus more on vicarious forms of learning—learning from experienced conspecifics—rather than simultaneous group learning. Instead, CCM seeks to explain how collective learning emerges from individual learners. Second, other theories, including Kao *et al*. [63], describe how groups of animals aggregate their collective knowledge and subsequently learn from these outcomes, neglecting the possibility that animals may learn to associate the actions of individual conspecifics and their outcomes. Additionally, the emphasis of such models on consensus decision-making limits their ability to generalize to organisms, such as humans, that may learn collectively but often behave divergently. To fill these gaps, CCM provides a highly flexible framework that explains how organisms learn as a group and display collective intelligence.

Various hypotheses regarding collective intelligence and learning in nature emerge from CCM. Consider, for instance, foraging in a stable world, where resources renew at fixed rates, versus in an unstable world, where renewal rates change over time. In a stable world, identifying the richest patch and determining the optimal dwelling time in that patch is a relatively simple task that an animal may efficiently solve alone [70,71]. This is, indeed, the world that is typically provided to animals when testing models of learning, choice and optimal foraging in the laboratory [72]. In such a world, CCM suggests that conspecifics do not provide useful guidance for foraging activities. Foraging interactions among conspecifics in a stable world may be reduced to mere competition for scarce resources [73]. In nature, however, resources rarely renew at stable rates. Seasonal changes, tidal fluctuations, migratory patterns, predator–prey dynamics and a host of other processes are likely to change the location of rich patches from time to time. In an unstable world, as rich patches become unexpectedly depleted and previously depleted patches become richer, predators benefit from learning to ignore navigational cues associated with the former and attend to those associated with the latter. In an unstable world, the CCM model suggests that conspecifics not only provide informative guidance for foraging activities, but such guidance persists even when instructive conspecifics are no longer around. Moreover, CCM does not require a sophisticated or specialized cognitive mechanism for collective intelligence to emerge in a noisy world. It only demands the application of a simple and well-conserved learning mechanism to the behaviour of conspecifics.

## Data Availability

This paper contains simulated data. The code and corresponding data have been uploaded as supplementary materials [74].
